# Advanced in immunological monitoring of HIV infection: profile of immune cells and cytokines in people living with HIV-1 in Benin

**DOI:** 10.1186/s12865-024-00615-1

**Published:** 2024-04-20

**Authors:** Yaou Pierrot Assogba, Adefounke Prudencia Adechina, Edmond Tchiakpe, Odilon Paterne Nouatin, René K. Kèkè, Moussa Bachabi, Honoré Sourou Bankole, Akadiri Yessoufou

**Affiliations:** 1 Laboratory of Cell Biology, Physiology and Immunology, Department of Biochemistry and Cellular Biology, Faculty of Sciences and Technology (FAST), Institute of Applied Biomedical Sciences (ISBA), University of Abomey-Calavi (UAC), Cotonou, 01 BP 526 Benin; 2 National Reference Laboratory of Health Program Fighting Against AIDS in Benin (LNR/PSLS), Ministry of Health, Cotonou, BP 1258 Benin; 3 Institut de Recherche Clinique du Bénin, Abomey-Calavi, Bénin; 4 The Laboratory of Research and Applied Biology (LARBA), Unité de Recherche en Microbiologie Appliquée et Pharmacologie des Substances Naturelles, EPAC, Université d’Abomey-Calavi (UAC), Cotonou, 01 BP 2009 Bénin; 5 Centre de Recherche pour la lutte contre les Maladies Infectieuses Tropicales (CReMIT), Université d’Abomey-Calavi (UAC), Cotonou, 01 BP 526 Benin; 6 Institute of Applied Biomedical Sciences (ISBA), Ministry of High Education and Scientific Research, Cotonou, 01 BP 918 Bénin

**Keywords:** PLHIV-1, Immune cells, Inflammatory cytokines, Therapeutic monitoring, Benin

## Abstract

**Background:**

Immune cells and cytokines have been linked to viremia dynamic and immune status during HIV infection. They may serve as useful biomarkers in the monitoring of people living with HIV-1 (PLHIV-1). The present work was aimed to assess whether cytokines and immune cell profiles may help in the therapeutic follow-up of PLHIV-1.

**Methods:**

Forty PLHIV-1 in treatment success (PLHIV-1s) and fifty PLHIV-1 in treatment failure (PLHIV-1f) followed at the University Hospital of Abomey-Calavi/Sô-Ava in Benin were enrolled. Twenty healthy persons were also recruited as control group. Circulating cytokines and immune cells were quantified respectively by ELISA and flow cytometry.

**Results:**

PLHIV-1 exhibited low proportions of CD4 + T cells, NK, NKT, granulocytes, classical and non-classical monocytes, and high proportions of CD8 + T cells, particularly in the PLHIV-1f group, compared to control subjects. Eosinophils, neutrophils and B cell frequencies did not change between the study groups. Circulating IFN-γ decreased whereas IL-4 significantly increased in PLHIV-1s compared to PLHIV-1f and control subjects even though the HIV infection in PLHIV-1s downregulated the high Th1 phenotype observed in control subjects. However, Th1/Th2 ratio remained biased to a Th1 phenotype in PLHIV-1f, suggesting that high viral load may have maintained a potential pro-inflammatory status in these patients. Data on inflammatory cytokines showed that IL-6 and TNF-α concentrations were significantly higher in PLHIV-1s and PLHIV-1f groups than in control subjects. Significant high levels of IL-5 and IL-7 were observed in PLHIV-1f compared to controls whereas PLHIV-1s presented only a high level of IL-5. No change was observed in IL-13 levels between the study groups.

**Conclusion:**

Our study shows that, in addition to CD4/CD8 T cell ratio, NK and NKT cells along with IL-6, TNF-α, IL-5 and IL-7 cytokines could serve as valuable immunological biomarkers in the therapeutic monitoring of PLHIV-1 although a larger number of patients would be necessary to confirm these results.

## Background

Antiretroviral (ARV) drugs block viral replication and are associated with reduced rates of HIV-1-related morbidity and mortality [1]. Several countries with limited resources have made efforts to increase the accessibility of ARVs to people living with HIV-1 (PLHIV-1) by following standardized treatment regimens [2, 3]. Currently, the plasma viral load and the number of CD4 + T lymphocytes are the conventional biological markers that allow for assessing the effectiveness of antiretroviral treatment in PLHIV-1. However, quantification of plasma viral load is the best way to assess disease progression and viral replication in the body responsible for the destruction of CD4 + T [4, 5]. The World Health Organization recommended these biological parameters as standard markers for monitoring PLHIV-1 on antiretroviral therapy [6]. However, these parameters remained difficult to access for the populations of countries with limited resources, for several reasons, in particular the high cost, the non-functionality of the measuring equipment and the centralization of these in the reference laboratories [7]. The advent of so-called “points of care” equipment has made it possible to overcome this difficulty by facilitating access to CD4 + T lymphocytes count for patients in the most remote regions of countries [8]. However, CD4 + T cell count is not always correlated with plasma viral load [7].

In the pathophysiology of HIV-1 infection, characteristic complex mechanisms result in a disturbance of immune cell the function, in particular that of CD4 + T helper lymphocytes and CD8 + cytotoxic T cells [9]. These immune-pathological events also contribute to the increased production of cytokines that may play central role in the pathogenesis of HIV-1 infection and modulate viral replication control and then the progression of HIV-1 pathology [10].

While some studies have established a negative correlation between CD4 + T cells, IL-2, IFN-γ lymphocytes and plasma viral load in PLHIV-1 on ART [11], other studies have shown that high plasma levels of IL-4 and IL-10 were correlated with a high plasma viral load concomitant with low levels of CD4 + T cells [12]. Study have highlighted the effect of plasma viral load on the extent of cytokine disruption showing that when cytokines were less affected, plasma viral load remained lowered in PLHIV-1 on antiretroviral therapy [13]. A strong positive correlation has been found between pro-and anti-inflammatory cytokines and plasma viral load in Africans infected with HIV-1 [14]. Plasma viral load offers a better predictive value of cytokine levels [14]. The kinetics of cytokines seems to be related to the dynamics of the viral and immune status of PLHIV-1. All these results suggest that cytokines along with immune cells may contribute as valuable additional parameters for the effective monitoring of viral progression in PLHIV-1.

Indeed, several studies have reported that Th1/Th2 cytokine balance may modulate HIV infection and may affect viral replication [15, 16]. Decreases in IL-2 and IFN-γ and concomitant increases in IL-4 and IL-10 would be associated with a decrease of antigen-specific immune response to HIV-1 infection [17]. Thus, cytokines may be considered as valuable markers of progression of AIDS. Therefore, the aim of this study was to assess whether immune cells and cytokines in PLHIV-1 would be helpful in the therapeutic monitoring of PLHIV-1.

## Subjects and methods

### Study population

The present study was conducted at the University Hospital of Abomey-Calavi/Sô-Ava in Benin. A total of ninety PLHIV-1 under two therapeutic lines were recruited including forty persons with viral load < 40 copies/ml (successful treatment group) and fifty persons with viral load ≥ 1000 copies/ml (treatment failure group). They are all under the medical monitoring of specialist clinicians for at least 06 months and under the first line treatment (TDF + 3TC + EFV or TDF + 3TC + DTG). Twenty healthy participants negative for HIV infection were also recruited and considered as control group. The later were not on any anti-inflammatory drug treatment and their blood count appeared normal and showed no signs of common infections (syphilis, hepatitis B and C) or abnormality. The other general inclusion criteria were as follow: participants should be at least 18 years old. Subjects should not be infected with HIV-2 or co-infected with HIV-1 and 2 or not having a history of diabetes mellitus, or not suffering from opportunistic infections such as toxoplasmosis, pneumocystosis, cryptococcosis, cytomegalovirus and Kaposi’s sarcoma, or in a state of pregnancy or under anti-inflammatory drugs.

### Sample collection and storage

At inclusion, 2.5 ml of venous whole blood was collected in both EDTA and dry tubes at the study sites and transported to the laboratory in refrigerated coolers. 300 µl of whole blood was systematically used for cell phenotyping. Plasma, isolated after centrifugation at 3500 rpm for 20 min, was frozen, stored at -80 °C until used for determination of plasma viral load at the Reference Laboratory of the National AIDS Health Program. Serum was obtained from blood collected in dry tube and used for cytokines quantification.

### Plasma viral load measurement

Plasma HIV-1 RNA viral load (VL) was performed using Cobas® TaqMan® 96/Cobas® Ampliprep® (CAP/CAP-CTM) HIV-1 quantitative assay (Roche Molecular Diagnostics, Basel, Switzerland), according to the manufacturer’s instructions and as previously described [18]. All samples from patients with viral load greater than 3 log were laboratory confirmed after patients received adherence counseling from physicians.

### Cell immunophenotyping

**Fig. 1:**
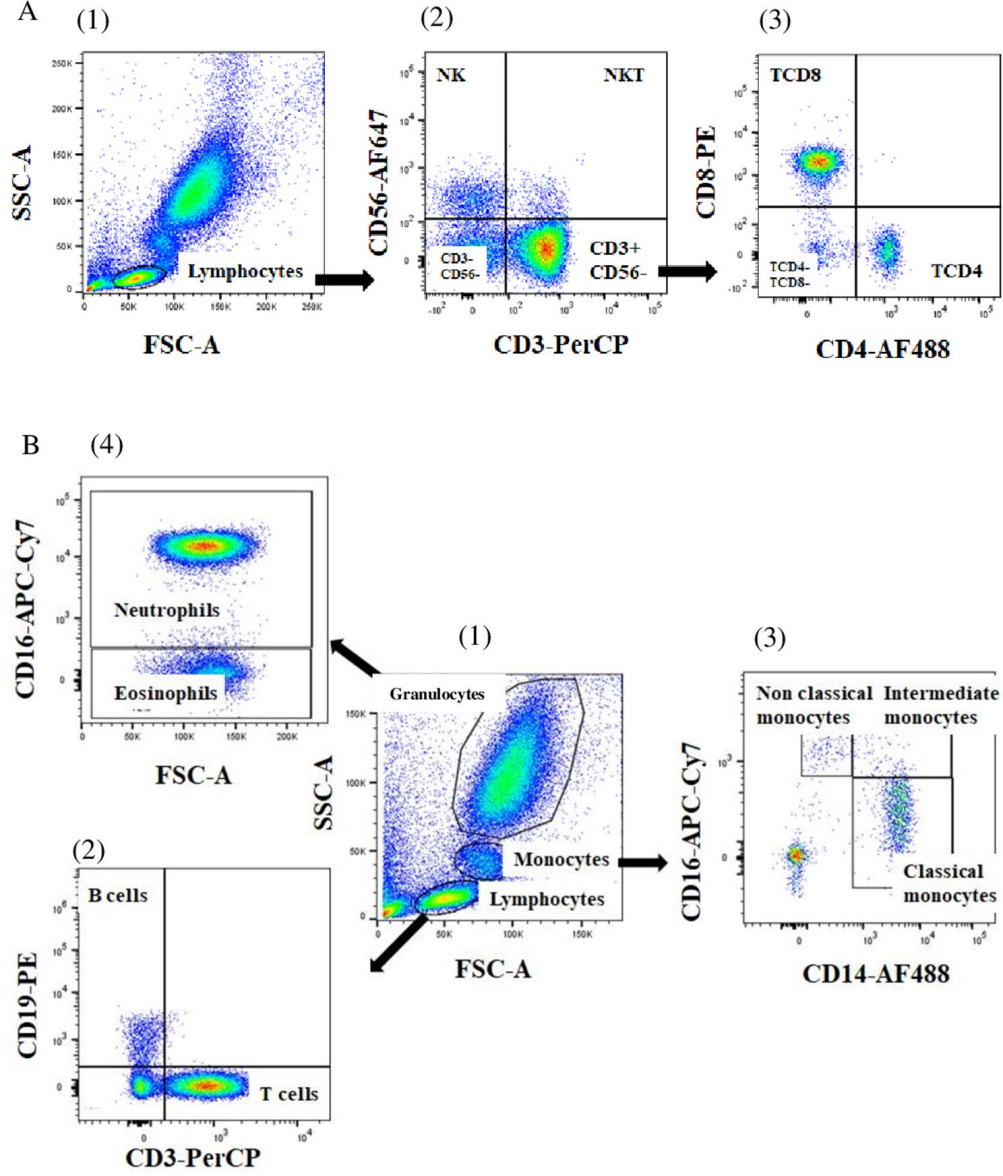
Cytometry-based gating strategies for leukoocytes’ subpopulations: T cells, B cells, monocytes, granulocytes and NK cells. A (1): Total lymphocytes’ population obtained based on SSC and FSC parameters. A (2): NK cells (CD3-CD56+) and NKT cells (CD3+CD56+) obtained by gating from A (1). A (3): CD4+ and CD8+ T cells obtained from CD3+CD56- T cells. B (1): Lymphocyte, monocyte and granulocyte populations were gated based on FSC-A and SSC-A in blood cells. B (2): Total B cells (CD19+) were gated from total lymphocytes using CD3 and CD19 monoclonal antibodies. B (3): Non-classical monocytes (CD16++CD14+), intermediate monocytes (CD14++CD16+), and classical monocytes (CD14++CD16-) were gated from total monocytes using CD14 and CD16 monoclonal antibodies. B (4): Neutrophil and eosinophil cells were identified based on CD16 in polymorphonuclear cells. SSC: Side scatter; FSC: Forward scatter; APC = allophycocyanin, PerCP = peridinin chlorophyll a protein; CD: Cluster of differentiation

The immune cells were stained as described elsewhere [19]. Briefly, immune cell phenotyping was carried out with monoclonal antibodies added into the EDTA-tube containing whole blood. Indeed, freshly collected whole blood (300 µl) was incubated with a mixture of monoclonal antibodies (5 µl per each) for 30 min at 4 °C in the dark. Then red blood cells were lysed using 2 mL Facs-Lysing buffer-1X added to each tube and the mixture was incubated for 10 min at room temperature in the dark and washed twice with PBS-1 × (3 mL of 1X PBS was added and centrifuged for 8 min at 1000 rpm at 4 °C). Optimal combinations of antibodies were used to identify specific immune cell subpopulations: CD3-PerCP, CD4-FITC, CD8-PE, CD-56APC monoclonal antibodies were used for identification of lymphocytes’ subpopulations (CD4 + T, CD8 + T, NK and NKT cells); CD3-PerCP, CD14-FITC, CD16-APC/Cy7 and CD19-PE monoclonal antibodies were used to label monocytes subpopulations, B cells lymphocytes and polymorphonuclear neutrophils and eosinophils. Stained cells were then suspended in 300 µl of PBS 1X and 100,000 cells from each tube were acquired with the BDFACSCanto II flow cytometer (BD Pharmingen, France). Flow cytometry data were analyzed using (FlowJo 7.6 software version 10.8.1, BD Pharmingen, France). The Fig. 1 shows the gating strategy for different cell subpopulation identification.

### Serum cytokine measurements

Serum concentrations of a set of cytokines were determined as we described elsewhere [20] by ELISA using Human Peprotech Elisa Kits (Rocky Hill, NJ 08553 USA) according to the manufacturer’ instructions. We determined T helper cell differentiation cytokines classified as Th1 (IFN-γ) and Th2 (IL-4) cytokines. TNF-α, IL-6 and IL-7 (as pro-inflammatory cytokines) and IL-5 and IL-13 (as anti-inflammatory cytokines) were also determined.

### Data analysis

Statistical analyzes were performed using R software (version 4.1.2). The graphs were made using the GraphPad software (version 8.3.0). The comparison of cytokine concentrations and cell frequencies, the IFN-γ/IL4 ratio between the different groups was made by the Kruskal-Wallis test with Dunn’s multiple comparison tests.

## Results

### Characteristics of the study population

As shown in Table 1, the median age of participants was 41 [35.75- 48.00] years. No significant difference was observed in the median age between different study groups (*p* = 0.21). The median viral load in PLHIV-1 with treatment failure was 4.56 [3.9–5.1] Log, the therapeutic lines were TDF + 3TC + EFV or TDF + 3TC + DTG. At the inclusion date, the highest rate of treatment failure was observed in patients under TDF + 3TC + EFV (70.83%). The average CD4 + T cells count was 301 [205–508.5] cells/µl in PLHIV-1 with successful treatment whereas it was 269 [176.3–433] cells/µl among patients with therapeutic failure. The average treatment duration with ARV was 60 [23–196] months in PLHIV-1 with successful treatment whereas it was 62 [14–192] months among patients with therapeutic failure (Table 1).

**Table 1:** Sociodemographic and clinical data of the control subjects (*n* = 20), people living with HIV-1 with therapeutic success (PLHIV-1s, *n* = 40) and people living with HIV-1 with therapeutic failure (PLHIV-1f, *n* = 50). Healthy control subjects and PLHIV-1 under ARV treatment for at least 6 months were recruited at the University hospital of Abomey-Calavi/Sô-Ava between October 2021 and April 2022

Characteristics	Healthy controls	PLHIV-1s	PLHIV-1f	Total number or median for all PLHIV-1	*p*-value
**Number**	20	40	50	110	-
**Sex**					
Female, n (%)	9 (45)	28 (70)	37 (74)	74	-
Male, n (%)	11 (55)	12 (30)	13 (26)	36	-
**Median age** [IQR]	39.5 [35.25–43]	45 [36.25–50.50]	40 [32.75–45.25]	41 [35.75–48.00]	^a^*p* = 0.21
**Median viral load (Log)** [IQR]	ND	U	4.56 [3.9–5.1]	4.56 [3.9–5.1]	-
**ARV treatment**					
TDF + 3TC + EFV, n (%)	-	15 (37.5)	37 (74)	52	-
TDF + 3TC + DTG, n (%)	-	25 (62.5)	13 (26)	38	-
**Median CD4 + T cell count (cells/µl)** [IQR]	ND	301 [205–508.5]	269 [148–417.5]	283 [176.3–433]	^b^*p* = 0.21
**ARV Treatment duration (months) [IQR]**	-	60 [23–196]	62 [14–192]	-	^−^

ND: Not determinated, U: Undetectable. TDF: Tenofovir, 3TC: lamivudine, EFV: Efavirenz, DTG: Dolutegravir, PLHIV-1: People living with HIV-1, IQR: Interquartile, ART: Antiretroviral. The statistical differences were determined using the nonparametric Kruskal-Wallis test and Mann-Whitney test. Differences were considered significant when p values are less than 0.05

^a^Kruskal-Wallis test between healthy controls, PLHIV-1s and PLHIV-1f groups. ^b^Mann-Whitney test between PLHIV-1s and PLHIV-1f groups

### Lymphocytes’ frequencies in the study groups

**Fig. 2:**
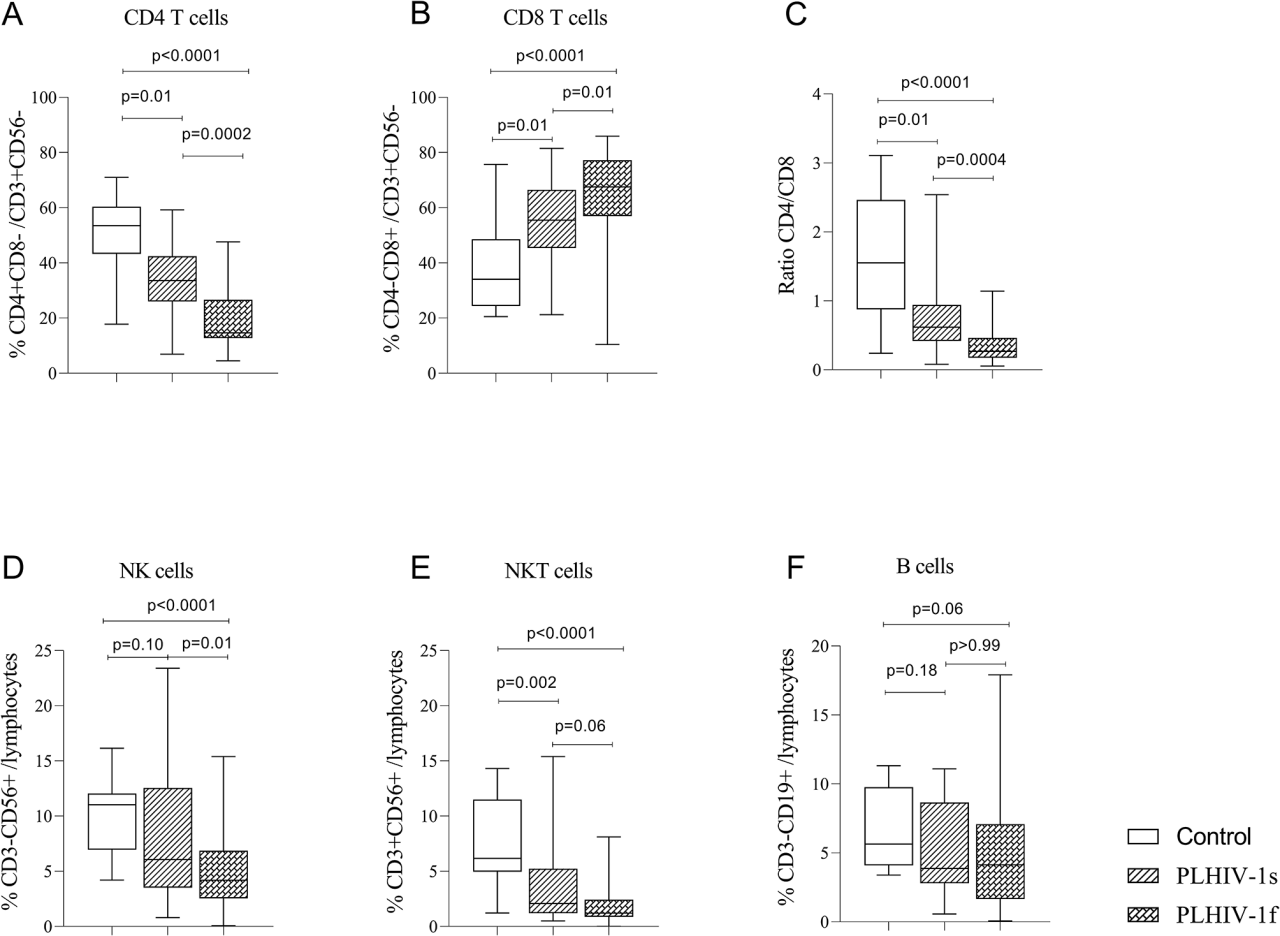
Frequencies of lymphocytes’ subpopulations and CD4/CD8 T cell ratios. The Figure shows the frequencies of CD4 T cells (2A), CD8 T cells (2B), ratio CD4/CD8 (2C), NK cells (2D), NKT cells (2E) and B cells (2F) in the control subjects (*n*=20), therapeutic success (*n*=40) and therapeutic failure (*n*=50) groups. Healthy control subjects and PLHIV-1 under ARV treatment for at least 6 months were recruited at the University hospital of Abomey-Calavi/Sô-Ava between October 2021 and April 2022. Data shown as box plots representing medians (with 25th and 75th percentiles). The statistical differences were determined using the nonparametric Kruskal-Wallis test with Dunn’s multiple comparisons. Differences were considered significant when p values are less than 0.05. NK: natural Killer, NKT: Natural Killer T, CD: Cluster of differentiation

As expected, the frequencies of CD4 + T cells were lower in both PLHIV-1s (*p* = 0.01) and PLHIV-1f (*p* < 0.0001) groups compared to the control subjects. Likewise, the frequency of CD4 + T cells was significantly lower in the PLHIV-1f group compared to the PLHIV-1s group (*p* = 0.0002) (Fig. 2A).

In opposite to CD4 + T cell profile, the frequencies of CD8 + T cells were higher in PLHIV-1s (*p* = 0.01) and in PLHIV-1f (*p* < 0.0001) as compared to the control subjects. Besides, the frequency of CD8 + T cells was higher in PLHIV-1f compared to PLHIV-1s (*p* = 0.01), (Fig. 2B). When comparing the CD4/CD8 ratio in the study groups, the results showed a significantly low CD4/CD8 ratio in PLHIV-1s and PLHIV-1f compared to control subjects (*p* = 0.01, *p* < 0, 0001, respectively). Specifically, the CD4/CD8 ratio was lower in PLHIV-1f compared to the PLHIV-1s group (*p* = 0.0004), Fig. 2C.

As shown in Fig. 2D, the frequency of NK cells was lower in PLHIV-1f compared to the control group (*p* < 0.0001) and to the PLHIV-1s group (*p* = 0.01). Same observation was made with NKT cells (Fig. 2E), with significant low frequencies in PLHIV-1s and PLHIV-1f compared to the control subjects (*p* = 0.002, *p* < 0.0001 respectively). No significant difference was observed in the frequencies of B cells between the study groups (Fig. 2F).

### Frequencies of monocytes’ subpopulations in the study groups

**Fig. 3:**
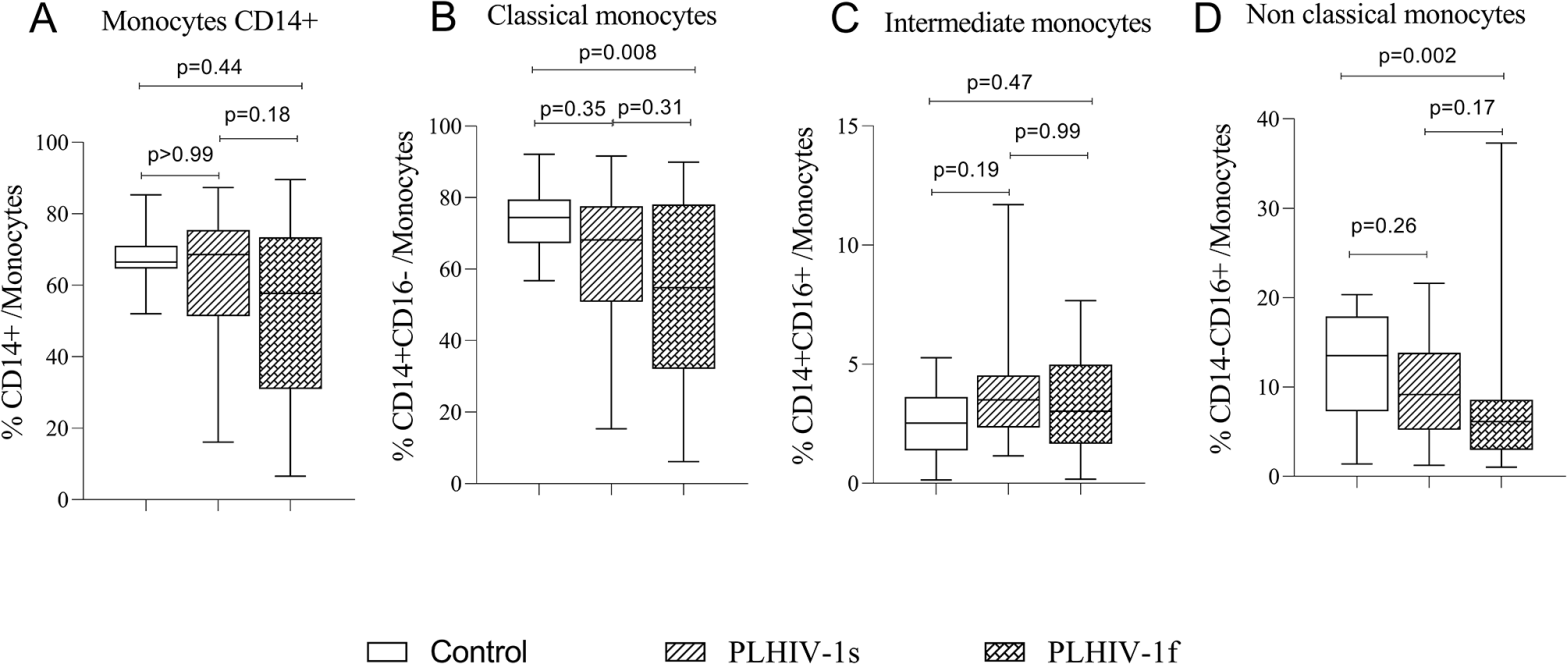
Frequencies of monocytes’ subpopulations in the study groups. The Fig. 3 shows the frequencies of total CD14+ monocytes (3A), classical (3B), intermediate (3C) and non-classical (3D) monocytes, in the control subjects (*n*=20), PLHIV-1 with therapeutic success (*n*=40) and PLHIV-1 with therapeutic failure (*n*=50) groups. Healthy control subjects and PLHIV-1 under ARV treatment for at least 6 months were recruited at the University hospital of Abomey-Calavi/Sô-Ava between October 2021 and April 2022. Data were shown as box plots representing medians (with 25th and 75th percentiles). The statistical differences were determined using the nonparametric Kruskal-Wallis test with Dunn’s multiple comparisons. Differences were considered significant when p values are less than 0.05

No significant difference was observed in the frequencies of total CD14 + monocytes (Fig. 3A) and intermediate monocytes between the study groups (Fig. 3C). However, the results showed significant low frequencies of classical monocytes (Fig. 3B, *p* = 0.008) and non-classical monocytes (Fig. 3D, *p* = 0.002) in therapeutic failure group compared to the control group.

### Frequencies of polymorphonuclear cells in the study groups

**Fig. 4:**
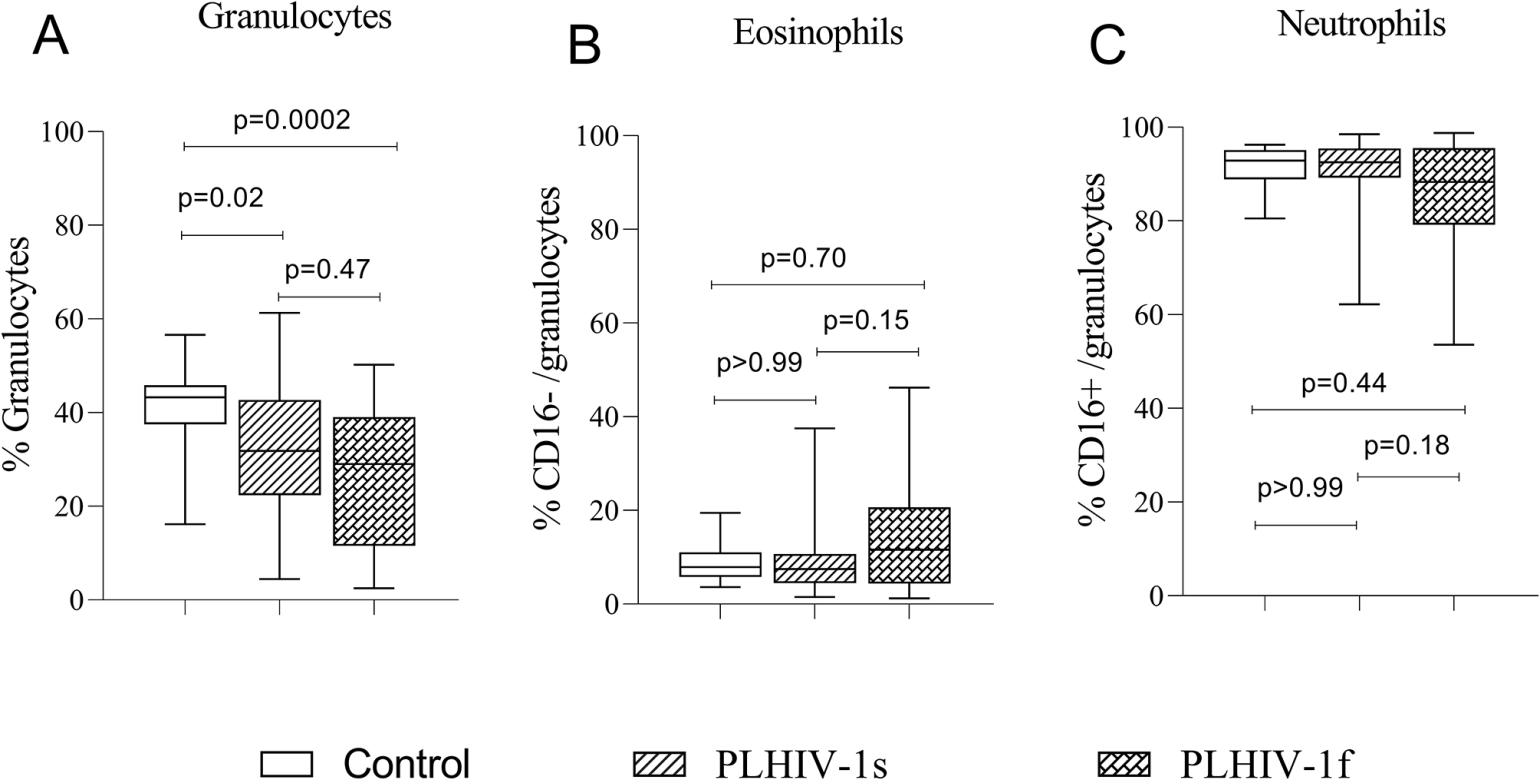
Frequencies of polymorphonuclear cells in the study groups. The Fig. 4 shows the frequencies of total granulocytes (4A), eosinophils (4B) and neutrophils (4C) in the control subjects (*n*=20), PLHIV-1 with therapeutic success (*n*=40) and PLHIV-1 with therapeutic failure (*n*=50). Healthy control subjects and PLHIV-1 under ARV treatment for at least 6 months were recruited at the University hospital of Abomey-Calavi/Sô-Ava between October 2021 and April 2022. Data were shown as box plots representing medians (with 25th and 75th percentiles). The statistical differences were determined using the nonparametric Kruskal-Wallis test with Dunn’s multiple comparisons. Differences were considered significant when p values are less than 0.05

The frequencies of granulocytes cells significantly decreased in PLHIV-1s (*p* = 0.02) and PLHIV-1f (*p* = 0.0002) (Fig. 4A) compared to the control groups. No significant difference was observed on eosinophils and neutrophils frequencies (Fig. 4B and C).

### Cytokine levels in study groups

We observed a significant low concentration of IFN-γ in PLHIV-1s compared to the control subjects (*p* < 0.0001) and to PLHIV-1f (*p* < 0.0001). TNF-α concentration was higher in both HIV-1 infected groups but remained statistically significant in PLHIV-1f group compared to the control subjects (*p* = 0.0007). The concentration of IL-6 in PLHIV-1s and PLHIV-1f groups were higher than that in the control group (*p* = 0.006, *p* < 0.0001 respectively). Indeed, IL-6 level was higher in PLHIV-1f compared to PLHIV-1s (*p* = 0.0003). On the other hand, we observed that PLHIV-1s exhibited a high concentration of IL-4 compared to the control subjects and PLHIV-1f (*p* = 0.02, *p* = 0.006 respectively). IL-5 cytokine concentration was higher in PLHIV-1s and PLHIV-1f compared to the control (*p* = 0.0006, *p* < 0.0001) whereas IL-7 levels significantly increased in only PLHIV-1f group compared to the control (*p* = 0.001). No significant difference was observed in the levels IL-13 between the study groups (Table 2).

**Table 2:** Plasma cytokine concentrations in study groups

Cytokines, Median, (IQR)	Healthy controls *N* = 20	PLHIV-1s *N* = 40	PLHIV-1f *N* = 50
**TNF-α (pg/ml)**	1085 (1047–1306)	1204 (1112–2211)	1511^**a**^ (1323–2627)
**IL-6 (pg/ml)**	121.5 (0,000–211.3)	524.7^**a**^ (162.2–918.5)	1364^**a, b**^ (820.5–2549)
**IL-5 (pg/ml)**	19.82 (11.08–29.66)	194.7^**a**^ (124.3–266.4)	176.9^**a**^ (21.3–250.2)
**IL-13 (pg/ml)**	932.3 (817.6–1224)	845.1 (583.9–1348)	955.8 (205.7–1768)
**IL-7 (pg/ml)**	209.2 (197.4–389.1)	295.3 (243.8–415.1)	547.6^**a**^ (311.5–1174)
**IFN-γ (pg/ml)**	1363 (1152–1813)	422.8^**a**^ (226.9–834.1)	1126^b^ (599.4–1625)
**IL-4 (pg/ml)**	135.3 (33.78–139.6)	365.5^**a**^ (101.5–492.7)	106.5^b^ (78–144.7)
**IFN-γ/IL-4** **(Th1/Th2)**	13.13 (8.25–47.53)	1.44^**a**^ (0.48–4.08)	7.31^b^ (1.50–22.43)

Plasma cytokine concentrations in study groups were assessed in the control subjects (*n* = 20), people living with HIV-1 with therapeutic success (PLHIV-1s, *n* = 40) and people living with HIV-1 with therapeutic failure (PLHIV-1f, *n* = 50). n = number of participants in each group. Healthy control subjects and PLHIV-1 under ARV treatment for at least 6 months were recruited at the University hospital of Abomey-Calavi/Sô-Ava between October 2021 and April 2022. IL: Interleukin. The statistical differences were performed using the nonparametric Kruskal-Wallis test with Dunn’s multiple comparison tests. IQR = interquartile range, pg/ml = picogram per milliliter. Differences were considered significant when p values are less than 0.05

^a^*p* < 0.05 PLHIV-1 group vs. healthy controls; ^b^*p* < 0.05 PLHIV-1s vs. PLHIV-1f

## Discussion

Therapeutic monitoring of PLHIV-1 constitutes a major challenge in the success of the fight against HIV infection. Any easy to access biological parameter that can help to improve this monitoring would be beneficial for this issue. In this context, we undertook this study to assess whether immune cells and cytokine would contribute to facilitate the therapeutic monitoring of PLHIV-1.

CD8 + T cells are known as cytotoxic cells promoted by cytokines mediated by CD4 + T type Th1 lymphocytes. In the present study, a significant increase in the percentage of CD8 + T lymphocytes was respectively observed in subjects with treatment failure. This observation could be explained by chronic immune activation and persistence of inflammation in subjects who failed treatment [21]. CD4 + T lymphocytes being the target of HIV, the replication of the latter obviously leads to the depletion of CD4 + T cells. This clearly justifies the negative correlation observed between the values of CD4 + T cells and the viral load reported in many studies [22, 23].

It has been proven that the number of NK and NKT cells could serve as predictor of HIV-1 disease progression [24, 25]. NK cells play an important role in the control of viral infections. They can indirectly eliminate infected cells through antibodies or directly by cellular cytotoxicity [26]. However, data are controversial on NK cell proportion in HIV infection. One study reported that even after partial restoration of NKs following ART, disturbances in their distribution and function persist [27]. Other studies revealed that during chronic HIV-1 infection, NK cells decreased significantly [28, 29]. NKT cells are at the frontier of innate and adaptive immune responses, and play a crucial role in HIV infection [30]. In the present study, the observed decrease of NKT cells in PLHIV-1f could be easily explained by the cell lysis [30]. In fact, it has been reported that under the action of the *Nef* gene, the truncated CD1d protein expressed on the surface of the APCs is eliminated by the NKT cells which then bind to the CD1d glycoproteins thanks to their receptor, thus causing their own lyses [30].

Human blood monocytes are classified into three subpopulations namely classical monocytes (CD14 + + CD16–), intermediates (CD14 + + CD16+) and non-classical (CD14 + CD16++) [31, 32]. CD16 + monocytes have mainly been reported in HIV-1 infected patients with high viremia [33, 34]. In our study classical and non-classical monocyte frequencies were decreased in PLHIV-1f probably linked to the destruction of these cells by HIV-1 [35, 36].

Granulocytes secrete biologically active compounds when they accumulate at sites of inflammation. These lysosomal substances are delivered to the tissues both by exocytosis of cytoplasmic granules and by metabolic reactions [37]. HIV causes granulocyte apoptosis in the bone marrow like ARVs, leading to granulocyte depletion [38, 39], as in the case of our study. On the contrary, ART would inhibit neutrophil apoptosis by promoting their recovery. This would explain the results of our study where no significant difference was observed in the subjects in failure and success compared to the control subjects [40]. Granulocytes in their terminal stage of differentiation transform into eosinophils. The latter are not uncommon in PLHIV-1 and are often observed early in the disease or at an advanced stage of the disease [41, 42]. The most common clinical manifestation associated with eosinophilia in PLHIV is a skin rash [42]. The fact that no difference was observed between the study groups showed that HIV infection did not influence the eosinophil frequencies in patients.

Like cells that already serve as biomarkers for monitoring treatment efficacy in PLHIV-1 under ART, we assessed plasma cytokine levels in PLHIV-1. It has been reported that persistent viral replication of HIV-1 in the tissues such as lymph nodes could lead to this increased level of IL-6 [43]. In the present study, we found that PLHIV-1 exhibited high levels of plasma IL-6 as compared to the control subjects. Moreover, the concentrations of this cytokine were elevated in failure compared success HIV-1 patients. These observations supported previous findings suggesting that elevated plasma levels of IL-6 that dramatically decrease during effective antiretroviral therapy may be a biological indicator of high viremia in PLHIV-1 on ARVs [44]. The other pro-inflammatory cytokine is TNF-α which hinders the entry of the virus into mononuclear phagocytes by preventing its interaction with the CCR5 coreceptor through the increase in the expression of chemokines that bind the CCR5 coreceptor such as RANTES [45]. The elevated levels of TNF-alpha in our study likely reflect protein stimulation of the gene encoding gp120, as evidenced by the ability of cycloheximide, a protein synthesis inhibitor, to block the secretion of TNF-α and IL-10 [46]. Besides, we observed that both pro-inflammatory cytokines, IL-6 and TNF-α, exhibited similar profiles in PLHIV-1. The levels of both cytokines increased in PLHIV-1 and their increases were more pronounced in PLHIV-1 in therapeutic failure. Therefore, the profile of both cytokines, in the present study, did not allow to decide on therapeutic outcome. However, their increase can testify to HIV infection.

Evidence from human studies have suggested that a shift between Th1 and Th2 cells may modulate the severity of AIDS [15, 16]. IFN-gamma is produced by Th1 cells which support cell-mediated immunity and as a consequence promote inflammation, cytotoxicity and delayed type hypersensitivity [47, 48]. IL-4 is produced by Th2 cells which promote humoral immunity and downregulate the inflammatory actions of Th1 cells [49]. Th1 cells also secrete IL-2, IFN-g and TNF-b, while Th2 cells secrete IL-4, IL-5, IL-6, IL-10 and IL-13 [50]. In the present study, IL-4 and IFN-γ exhibited opposite behaviors when we compared PVVIH-1s and PVVIH-1f to the control subjects. The first cytokine IL-4 increases while the second decreases in PVVIH-1. The observed low level of plasma IFN-γ could be explained by ART that blocking viral replication which would inhibit the synthesis of mRNA responsible for the production of IFN-γ as reported in Kenya [12]. However, other authors have reported elevated IFN- γ levels in HIV-1 infected patients as reported in USA [51, 52]. This observation could be explained by the fact that the CD4 + T lymphocytes infected by the viruses using the CXCR5 coreceptors do not fuse so well that each lymphocyte produces its interferon gamma [53].

In the present study, IL-4 secreted by activated CD4 + T cells would inhibit the spread of non-syncytium viruses using CCR5 coreceptors and increase the spread of HIV-1 isolates using CXCR4 and inducing syncytia. This may result in the low production of IL-4 observed in our study and an acceleration of the disease towards the AIDS phase [54]. Reduced levels of IL-4 have been reported in supernatants of cultured PBMCs and purified CD4 + T cells obtained from HIV-1 infected individuals [55].

To better appreciate the shift between Th1 and Th2 phenotype in PLHIV-1 under ART, we calculated the Th1/Th2 ratios between IFN-γ (Th1) and IL-4 (Th2) levels (Table 2). We found that Th1/Th2 ratio was highly polarized towards Th1 phenotype in the control subjects (13.13 time-fold) decreased sharply in PLHIV-1s in therapeutic success, suggesting that HIV infection strongly downregulated Th1 phenotype. However, in PLHIV-1f, the ratio remained biased to Th1 phenotype (7.31 time-fold), suggesting that high viral load may have maintained a potential pro-inflammatory status in these patients [51, 52].

HIV replication in the presence of ARVs is a sign of the presence of resistance mutations in which elevated levels of IL-5 have been reported [56] as in the case of our study. IL-7 is essential for T cell homeostasis. Its production increases as in our study as part of a homeostatic response due to T cell depletion [57]. IL-13 is produced by T cells and dendritic cells and inhibits virus infectivity by down-modulating CCR5 expression on monocyte-derived macrophages. A negative association between viral load and IL-13 secretion has been reported in PLVIH-1s [58]. But, IL-13 expression has been reported to be increased in infected patients and improve B cell growth and survival [59]. This observation could be in favor of the result reported in our study where non-significant difference has been observed.

## Conclusion

In summary, the present study contributes to enrich data on the immunological monitoring parameters of PLHIV-1. As commonly demonstrated, an increase in CD8 + T cells and a decrease CD4 + T cells were observed in PLHIV-1 with treatment failure. We also observed marked low frequencies of NK, NKT cells, classical and non-classical monocytes and total granulocytes in treatment failure group who also exhibited a high concentration of TNF-α, IL-5, IL-6 and IL-7. Interestingly, we noted that the low frequencies of NK and NKT cells and high levels of IL-6 and TNF-α were perfectly correlated with high viremia in PLHIV-1f under ARV in treatment failure. As the ratio of CD4/CD8 T cells has always been so far, the present study shows that NK and NKT cells as well as IL-6, TNF-α, IL-5 and IL-7 cytokines could be valuable additional immunological biomarkers in the therapeutic monitoring of PLHIV-1. Prospective studies including a larger number of HIV-1 infected patients would be necessary and recommended to confirm these results.

### Limitations

Our study did not lack limitations. Firstly, the declarations of the state of health of the control patients did not benefit from verification elements. Secondly, the sample size was small and did not allow definitive conclusions. Additionally, an intracellular cytokine staining which we did not perform would have been more informative and helpful to identify the cellular sources of cytokines.

## Data Availability

The datasets used and/or analyzed during the current study are available from the corresponding author on reasonable request.

## Abbreviations

ART: Antiretroviral therapy
HIV: Human immunodeficiency virus
PLHIV-1: People living with HIV-1
NRTI: Nucleoside reverse transcriptase inhibitors
NNRTI: Non-nucleoside reverse transcriptase inhibitor
IP: Protease inhibitor
IIN: Integrase inhibitor
TDF: Tenofovir
3TC: Lamuvidine
DTG: Dolutegravir
EFV: Efavirenz
IL: Interleukin
TNF-α: Tumor Necrosis Factor Alpha
IFN-γ: Interferon gamma
Th: T-helper
